# Anti-cN1A Antibodies Are Associated with More Severe Dysphagia in Sporadic Inclusion Body Myositis

**DOI:** 10.3390/cells10051146

**Published:** 2021-05-10

**Authors:** Matteo Lucchini, Lorenzo Maggi, Elena Pegoraro, Massimiliano Filosto, Carmelo Rodolico, Giovanni Antonini, Matteo Garibaldi, Maria Lucia Valentino, Gabriele Siciliano, Giorgio Tasca, Valeria De Arcangelis, Chiara De Fino, Massimiliano Mirabella

**Affiliations:** 1 Fondazione Policlinico Universitario Agostino Gemelli IRCCS, 00168 Roma, Italy; giorgiotasca81@gmail.com (G.T.); v.dearcangelis@gmail.com (V.D.A.); chiaradefino@me.com (C.D.F.); massimiliano.mirabella@unicatt.it (M.M.); 2 Department of Neurosciences, Section of Neurology, Università Cattolica del Sacro Cuore, 00168 Roma, Italy; 3 Neuroimmunology and Neuromuscular Diseases Unit, Fondazione IRCCS Istituto Neurologico Carlo Besta, 20133 Milano, Italy; Lorenzo.Maggi@istituto-besta.it; 4 Department of Neurosciences, University of Padova, 35122 Padova, Italy; elena.pegoraro@unipd.it; 5 Department of Clinical and Experimental Sciences, University of Brescia, NeMO-Brescia Clinical Center for Neuromuscular Diseases, 25121 Brescia, Italy; massimiliano.filosto@unibs.it; 6 Department of Clinical and Experimental Medicine, University of Messina, 98122 Messina, Italy; crodolico@unime.it; 7 Department of Neuroscience, Mental Health and Sensory Organs (NESMOS), School of Medicine and Psychology, Sant’Andrea Hospital, Sapienza University of Rome, 00189 Rome, Italy; vanni.antonini@gmail.com (G.A.); matteo.garibaldi@uniroma1.it (M.G.); 8 IRCCS Istituto delle Scienze Neurologiche di Bologna, 40139 Bologna, Italy; marialucia.valentino@unibo.it; 9 Department of Biomedical and Neuromotor Sciences, University of Bologna, 40126 Bologna, Italy; 10 Department of Clinical and Experimental Medicine, University of Pisa, 56126 Pisa, Italy; gabriele.siciliano@unipi.it

**Keywords:** inclusion body myositis, anti-cN1A antibodies, inflammatory myopathies, autoantibodies

## Abstract

In recent years, an autoantibody directed against the 5′-citosolic nucleotidase1A (cN1A) was identified in the sera of sporadic inclusion body myositis (s-IBM) patients with widely variable sensitivity (33%–76%) and specificity (87%–100%). We assessed the sensitivity/specificity of anti-cN1A antibodies in an Italian cohort of s-IBM patients, searching for a potential correlation with clinical data. We collected clinical data and sera from 62 consecutive s-IBM patients and 62 other inflammatory myopathies patients. Testing for anti-cN1A antibodies was performed using a commercial ELISA. Anti-cN1A antibodies were detected in 23 s-IBM patients, resulting in a sensitivity of 37.1% with a specificity of 96.8%. Positive and negative predictive values were 92.0% and 60.6%, respectively. We did not find significant difference regarding demographic variables, nor quadriceps or finger flexor weakness. Nevertheless, we found that anti-cN1A-positive patients presented significantly lower scores in IBMFRS item 1 (swallowing, *p* = 0.045) and more frequently reported more severe swallowing problems, expressed as an IBMFRS item 1 score ≤ 2 (*p* < 0.001). We confirmed the low sensitivity and high specificity of anti-cN1A Ab in s-IBM patients with a high positive predictive value. The presence of anti-CN1A antibodies identified patients with a greater risk of more severe dysphagia.

## 1. Introduction

Sporadic inclusion body myositis (s-IBM) is an idiopathic inflammatory myopathy (IIM) with peculiar histological and clinical features that include quadriceps and deep finger flexor weakness and often pharyngeal muscle impairment causing dysphagia [1]. Inflammatory infiltrates, mainly comprising CD8+ lymphocytes surrounding non-necrotic muscle fibers, along with degenerative features including autophagic abnormalities, such as eosinophilic inclusions and “rimmed” vacuoles, characterize muscle pathology [2]. s-IBM represents the most frequent cause of acquired myopathy after 50 years of age, and clinical onset rarely occurs before the fourth decade of life. Due to the slow progression course and the requirement of specific histopathological and clinical features for a definite diagnosis that can be absent in earlier stages of the disease, s-IBM is frequently misdiagnosed with a mean diagnostic delay of 5 years [3]. The 2011 European Neuromuscular Centre (ENMC) diagnostic criteria simplified the pathological criteria and underlined the importance of specific clinical features allowing an earlier diagnosis [4]. The 2011 ENMC diagnostic criteria are more sensitive but less specific when compared with the more stringent Griggs criteria [1]. Muscle magnetic resonance imaging (MRI) studies identified a characteristic pattern of muscle involvement in s-IBM patients. “Imaging criteria” have been proposed that include bilateral distal fatty replacements and STIR hyperintensities of the quadriceps with relative sparing of the posterior compartment of the thigh [5]. In 2011, an autoantibody directed against a 43 kD protein was identified in the sera of s-IBM patients [6]. Two years later, the target antigen was identified in the 5′-citosolic nucleotidase 1A (cN1A) [7,8]. The sensitivity of anti-cN1A antibodies in s-IBM significantly varies in different studies, ranging from 33% to 76%, mainly due to different detection methods and cut-off thresholds [9,10,11,12,13,14,15,16,17]. Although test specificity ranges from 87% to 100%, anti-cN1A antibodies were also found in the sera of patients with other IIMs and other autoimmune diseases (mainly systemic lupus erythematosus and Sjögren syndrome) [12,18]. The aim of the present study was to evaluate anti-cN1A antibody sensitivity and specificity in a large Italian cohort of s-IBM patients and to correlate the presence of antibodies with clinical features and disease severity.

## 2. Materials and Methods

### 2.1. Patients and Sample Collection

Clinical data and sera from 62 consecutive s-IBM patients attending eight Italian neuromuscular centers were prospectively collected to evaluate the presence of anti-CN1A antibody. Exclusion criteria were treatment with either intravenous human immunoglobulin (IVIG) or immunosuppressants in the last 6 months. Oral prednisone was permitted at a dosage exceeding 5 mg per day. All subjects underwent muscle biopsy and were diagnosed with clinicopathologically or clinically defined s-IBM, or probable s-IBM, based on the 2011 ENMC research diagnostic criteria [4]. We also tested serum samples of 62 patients with inflammatory myopathies other than s-IBM (all from the Fondazione Policlinico A. Gemelli IRCCS) as a control group to evaluate anti-cN1A antibody specificity. All data were gathered after approval from the local ethical committee and after obtaining informed consent from each participant.

### 2.2. Clinical Features

At the time of sample collection, the following features were recorded: age at onset, at diagnosis and at sample collection; gender; disease duration; creatine phosphokinase (CK) level (UI/L); symptoms at onset; Medical Research Council (MRC) strength grading for quadriceps muscles; presence of dysphagia and concurrent medical illnesses. Patients were also clinically evaluated with the s-IBM functional rating scale (IBMFRS) [19], which specifically evaluates the typical features of s-IBM patients, such as dysphagia, quadriceps and finger flexor digitorum weakness. The IBMFRS is a 10-point functional rating scale with each item graded from 0 (identifying the worst condition) to 4 (normal) with a maximum score of 40. The IBMFRS item 1 evaluates swallowing, and a score of 2 reflects significant dysphagia with the need of dietary consistency changes. Items 2 to 5 assess upper limb functioning. Item 5, together with items 6 and 7, evaluates domestic daily living activities, while items 8 to 10 score lower limb functioning.

### 2.3. Serological Testing for Anti-CN1A Antibodies

Testing for anti-cN1A autoantibodies was performed using a commercially available enzyme-linked immunosorbent (ELISA) method (Euroimmun, Lubeck, Germany) [20]. Briefly, wells coated with Euroimmun full-length cN1A antigens were incubated with diluted patient sera, and the bound antibodies were detected colorimetrically with horseradish peroxidase-conjugated with goat anti-human immunoglobulin G (IgG, Fab-specific). Positive serum samples were related to the level of measurement signal proportionate to the concentration of cN1A antibodies and can be determined using a single calibrator with known cN1A concentrations. Each test included a positive and a negative control provided by the manufacturer.

### 2.4. Statistical Analysis

All values are reported as the mean (±standard deviation) unless indicated otherwise. We compared clinical data of anti-cN1A-positive and -negative s-IBM patients. We used the t-test and the Kruskal–Wallis test for continuous variables and the chi-squared test for categorical variables. All two-tailed *p*-values <0.05 were considered as significant.

## 3. Results

In the s-IBM cohort, the mean age at onset was 67.3 years with a disease duration of 8.3 years and a male predominance (62.9%). The mean diagnostic delay (time from onset to diagnosis) was 5.0 years. Almost all patients were ambulant, and about half reported dysphagia. The most frequent presenting symptom was proximal lower limb weakness (74.2%). Most of the patients (41%–66.1%) had clinicopathologically defined IBM following the 2011 ENMC diagnostic criteria, while only seven patients had a diagnosis of probable IBM. The main clinical features of the s-IBM and IIM cohorts are summarized in Table 1 and Table 2, respectively.

An anti-cN1A test was positive in 23 out of 62 s-IBM patients, resulting in a sensitivity of 37.1%. Only two patients of the 62 IIMs, other than the s-IBM patients tested, were positive, with a specificity of 96.8%. Positive and negative predictive values were 92.0% and 60.6%, respectively.

We next compared patients’ characteristics between anti-cN1A antibody-positive and -negative patients (Table 3). We did not find differences in terms of age at onset or disease duration. Regarding the 2011 ENMC diagnostic criteria, we did not find significant differences in anti-cN1A antibody prevalence within the three diagnostic groups (clinicopathologically defined IBM 39.5%, clinically defined IBM 35.7% and probable IBM 28.6%, *p* = 0.863).

The IBMFRS composite score was lower in positive patients without reaching statistical significance. By analyzing each IBMFRS item, we only found a significant difference regarding item 1 (*p* = 0.04). This item explores the presence of swallowing problems, and its mean score was significantly lower in positive patients. Moreover, anti-cN1A antibody-positive patients were significantly more likely to have more severe swallowing problems, expressed as an IBMFRS item 1 score ≤ 2 (52.4% vs. 9.7% in the whole cohort, *p* < 0.001).

## 4. Discussion

In different published studies, anti-cN1A antibody sensitivity greatly varies, ranging from 30% to 70% [16]. This variability largely depends on the different detection methods and cut-off values for positivity. Anti-cN1A antibodies have a high specificity to distinguish s-IBM from other IIMs, although it can be detected in up to 20% of patients suffering from rheumatological diseases [12].

In the present study, we found a sensitivity of 37.1% using a commercially available ELISA test. Only a few studies used the same detection method with a sensitivity ranging between 35% and 50% [13,15,20,21]. Moreover, in the paper of Felice et al., the sensitivity dropped from 50% to 30% when considering only strongly positive patients [15]. We also confirmed the high specificity of anti-cN1A antibodies to distinguish s-IBM from other IIMs.

We decided to exclude patients with concurrent treatment with IVIG, immunosuppressant or high dose steroids to prevent a potential confounder for both clinical and serological evaluations. Despite there being no drugs with proven and lasting efficacy in s-IBM, there are some reports of transient clinical improvement following IVIG, immunosuppressants and steroid treatments [22,23,24]. Furthermore, drugs that modify the immune response could alter the eventual demonstration of anti-cN1A antibodies in the serum, potentially resulting in a lower sensitivity.

Diagnostic delay is still a substantial issue for s-IBM due to the slowly progressive course and the low sensitivity of classical pathological features in early stages, leading to lengthy and potentially damaging immunosuppressive treatments. In our study, the mean time from first symptoms to a definite s-IBM diagnosis was 5 years, consistent with published data [3,25]. The availability of a highly specific serum biomarker might contribute to reducing diagnostic delay.

Previous studies explored possible differences between anti-cN1A-positive and -negative s-IBM cohorts relative to clinical and pathological findings with inconsistent results. A small monocentric study demonstrated that anti-cN1A-positive patients presented a more severe phenotype with significant bulbar involvement [11]. In a large multicenter cohort, a higher risk of mortality, mainly due to pneumological complications, was associated with the presence of anti-cN1A antibodies [14]. Moreover, a recent study found that the age at onset was higher in anti-cN1A-positive patients [15].

In our study, we did not find differences regarding age at onset or disease duration. The IBMFRS scores were lower in Ab-positive patients without reaching statistical significance, but we found a significant difference regarding swallowing problems with lower IBMFRS scores and more severe dysphagia in Ab-positive-patients, also supporting a possible association between the presence of the antibody and severe bulbar dysfunction, as reported by Goyal et al. [11].

Swallowing difficulties are very common in s-IBM patients, with the reported incidence ranging from 40% to 80% [26,27]. This highly variable incidence could be related to the patient selection method and to the type of dysphagia assessment, since patients tend to underestimate this problem in the early phase of the disease [28]. Despite being more commonly reported as a late complication of the disease, dysphagia could represent the presenting symptom in some cases [29]. Compared to other IIMs, s-IBM patients had more frequent and severe dysphagia [30].

Different approaches, including a videofluoroscopic swallow study and a flexible endoscopic evaluation, have been studied to evaluate swallowing difficulties in this disease, but there is a lack of standardization of outcome measures to evaluate and monitor the evolution of dysphagia [31]. Several non-invasive and invasive treatment strategies were evaluated to manage swallowing difficulties. Non-invasive treatments include IVIG or subcutaneous immunoglobulin, while possible invasive approaches are the balloon dilation of the pharyngoesophageal segment, botulinum toxin injection to the cricopharyngeus muscle, cricopharyngeal myotomy and the use of a percutaneous endoscopic gastrostomy feeding tube [32].

The most severe complications of dysphagia are represented by aspiration leading to pneumonia and malnutrition with an increased risk of death in patients with more severe dysphagia [33,34].

Several questions are still unresolved regarding the relationship between anti-cN1A antibodies and s-IBM. First, there is a need for the standardization of detection methods and cut-off levels. Most studies have used a commercial or in-house ELISA as a detection method, and in recent years, a cell-based assay was also developed [13]. More recently, Amlani et al. developed an addressable laser bead immunoassay (ALBIA) using a full-length human recombinant protein demonstrating a strong concordance with a commercially available ELISA kit [17].

### Study Limits

The first partial limitation of the study is the relatively small cohort compared to the largest in the field. However, patients enrolled in those studies have a different genetic background than those enrolled in ours. The second relates to the fact that we evaluated dysphagia using subjective scores and not instrumental methods. Moreover, despite being widely used in s-IBM observational and pharmacological studies, IBMFRS has not yet been formally validated. Finally, treatment exclusion criteria could represent a selection bias, potentially excluding more severely affected patients.

## 5. Conclusions

We confirmed the low prevalence of anti-cN1A antibodies in s-IBM patients. However, anti-cN1A antibodies showed high specificity for the disease. We were also able to distinguish s-IBM from other IIMs. In our cohort, the presence of anti-cN1A antibodies was associated with more severe dysphagia.

## Figures and Tables

**Table 1:** Baseline characteristics of the s-IBM cohort.

	s-IBM *n* = 62
Female sex, *n* (%)	23 (37.1)
Age, years	67.3 (9.6)
Disease duration, years	8.3 (7.5)
Age at onset, years	59.0 (10.0)
Diagnostic delay, years	5.0 (4.0)
CK levels (UI/L)	675.5 (481.0)
Quadriceps MRC (0–5)	3.2 (1.3)
Dysphagia, *n* (%)	31 (50.0)
Ambulant patients, *n* (%)	58 (93.6)
2011 ENMC criteria	
Clinicopathologically defined IBM	41 (66.1)
Clinically defined IBM	14 (22.6)
Probable IBM	7 (11.3)
Symptoms at onset, *n* (%)	
Proximal lower limb weakness	46 (74.2)
Distal upper limb weakness	11 (17.7)
Dysphagia	5 (8.1)
IBMFRS score	27.4 (7.7)

All values are reported as mean (standard deviation) unless indicated otherwise. CK: creatine kinase; MRC: Medical Research Council; ENMC: European Neuromuscular Centre; s-IBM: sporadic inclusion body myositis; IBMFRS: s-IBM functional rating scale.

**Table 2:** Baseline characteristics of the IIM cohort.

	IIMs Other than s-IBM *n* = 62
Female sex, *n* (%)	38 (61.3)
Age, years	63.6 (14.0)
Inflammatory myopathy classification, *n* (%)	
Immune-mediated necrotizing myopathy	28 (45.2)
Dermatomyositis	20 (32.2)
Polymyositis	10 (16.1)
Overlap Myositis	4 (6.5)

All values are reported as mean (standard deviation) unless indicated otherwise. IIM: idiopathic inflammatory myopathy; s-IBM: sporadic inclusion body myositis.

**Table 3:** Comparison between anti-cN1A-positive and -negative s-IBM patients.

	Anti-cN1A Positive *n* = 23	Anti-cN1A Negative *n* = 39	*p* Value
Female sex, %	39.1	38.5	0.958
Age at onset, years	59.7 (9.8)	58.6 (10.2)	0.664
Diagnostic delay, years	5.3 (4.6)	4.8 (3.7)	0.640
Age, years	68.9 (8.0)	66.3 (10.4)	0.318
Disease duration, years	9.1 (6.4)	7.8 (5.8)	0.391
CK levels (UI/L)	782.0 (674.4)	606.6 (289.4)	0.185
Dysphagia, %	60.9	43.6	0.189
Ambulant patients, %	91.3	94.9	0.581
MRC quadriceps (range 0–5)	2.9 (1.4)	3.3 (1.2)	0.196
IBMFRS score (range 0–40)	25.9 (8.4)	28.4 (7.1)	0.253
IBMFRS item 1 (Dysphagia)	2.8 (1.1)	3.3 (0.9)	**0.045**
IBMFRS item 1 ≤ 2, % (whole cohort)	52.4	9.7	**<0.001**
IBMFRS item 2–5 (Upper limb)	11.2 (3.5)	12.4 (3.5)	0.199
IBMFRS item 8–10 (Lower limb)	5.9 (2.8)	6.6 (2.5)	0.381

All values are reported as mean (standard deviation) unless indicated otherwise. Significant differences at a two-sided α level <0.05 are displayed in bold. CK: creatine kinase; MRC: Medical Research Council; IBMFRS: sporadic inclusion body myositis functional rating scale.

## Data Availability

The data presented in this study are available on reasonable request from the corresponding author.
